# Implementing mobile information technology in clinical nursing education: how, why, when, where and what happened? Some answers from a review of the literature

**DOI:** 10.1186/1472-6963-14-S2-P88

**Published:** 2014-07-07

**Authors:** Siobhan O’Connor, Tom Andrews

**Affiliations:** 1 Health Information Systems Research Centre, Department of Accounting, Finance and Information Systems, University College Cork, Cork, Ireland; 2 Catherine McAuley School of Nursing and Midwifery, University College Cork, Cork, Ireland

## Background

Clinical practice presents a variety of challenges for nursing students which can impact their learning and application of knowledge and skills. Their inexperience coupled with the lack of supervision and ad hoc nature of learning in clinical environments can reduce their hands-on skills and negatively impact patient care. New methods are needed to help nursing students and educators address the theory-practice gap [1]. Information and communication technologies (ICT) such as mobile devices are being proposed as one way to support nursing students in clinical practice as they provide instant access to evidence based information at the point of care [2]. Despite the advantages it offers implementing mobile technology in clinical nursing education has proved challenging. The literature review aims to investigate how, why, when and where hand-held devices have been utilised in clinical nursing education and what factors facilitated or hindered their use.

## Materials and methods

Online bibliographical databases including CINAHL, ERIC, MEDLINE, PubMed and The Cochrane Library were searched using a combination of key terms such as: mobile, handheld, personal digital assistant, PDA, smarpthone, tablet computer, technology, nurs*, student, education, learning and training. Studies included in the review were primary research studies, published in English in peer reviewed journals between January 2000 and December 2013.

## Results

Of the 216 abstracts identified, 24 were included in the study. These articles highlight the homogeneity of mobile platforms currently in use, with personal digital assistants being the predominent device despite newer technologies being available. A variety of mobile applications and how they are used by nursing students in clinical practice is also summarised. Although a number of benefits to using mobile devices for clinical learning are identified these are limited by a multiplicity of socio-technical barriers.

## Conclusions

Mobile technology has spawned a cultural shift creating continuous and pervasive access to data. These unique features can support nursing student to improve their knowledge, skills and clinical practice. However many barriers to implementing mobile devices still need to addressed before they become integrated into routine nursing practice.
